# Recent advances in the understanding of Nipah virus immunopathogenesis and anti-viral approaches

**DOI:** 10.12688/f1000research.19975.1

**Published:** 2019-10-16

**Authors:** Rodolphe Pelissier, Mathieu Iampietro, Branka Horvat

**Affiliations:** 1 International Center for Infectiology Research-CIRI, Immunobiology of Viral Infections team, Inserm U1111, CNRS, UMR5308, University of Lyon, Ecole Normale Supérieure de Lyon, France

**Keywords:** Nipah virus, innate immunity, adaptive immunity, pathogenesis, animal models, contra-measures

## Abstract

Nipah virus (NiV) is a highly lethal zoonotic paramyxovirus that emerged at the end of last century as a human pathogen capable of causing severe acute respiratory infection and encephalitis. Although NiV provokes serious diseases in numerous mammalian species, the infection seems to be asymptomatic in NiV natural hosts, the fruit bats, which provide a continuous virus source for further outbreaks. Consecutive human-to-human transmission has been frequently observed during outbreaks in Bangladesh and India. NiV was shown to interfere with the innate immune response and interferon type I signaling, restraining the anti-viral response and permitting viral spread. Studies of adaptive immunity in infected patients and animal models have suggested an unbalanced immune response during NiV infection. Here, we summarize some of the recent studies of NiV pathogenesis and NiV-induced modulation of both innate and adaptive immune responses, as well as the development of novel prophylactic and therapeutic approaches, necessary to control this highly lethal emerging infection.

## Introduction

Emerging infectious diseases pose a significant threat to human and animal welfare in the world. Nipah virus (NiV) is a recently emerged zoonotic Paramyxovirus, from the *Mononegavirales* order, capable of causing considerable morbidity and mortality in numerous mammalian species, including humans ^1– 3^. Although NiV infection remains rare in humans, this virus has captured the attention of both scientific and public health communities because of its high fatality rate, ranging from 40% in Malaysia to more than 90% in Bangladesh and India, where it was associated with frequent person-to-person transmission ^4, 5^. Having the capacity to cause severe zoonosis with serious health and economic problems, without efficient treatment yet available, NiV is considered a possible agent for bioterrorism ^6^, has global pandemic potential ^7^, and is classified as a biosecurity level 4 (BSL4) pathogen. In 2015, the World Health Organization included NiV in the Blueprint list of eight priority pathogens for research and development in a public health emergency context ^8^. Furthermore, the Coalition for Epidemic Preparedness Innovations has targeted NiV as a priority for vaccine development on the basis of its high potential to cause severe outbreaks ^9^.

## Viral structure and epidemiology

NiV belongs to *Henipavirus* genus, along with the highly pathogenic Hendra virus (HeV), which emerged in Australia in 1994 ^10^, and the non-pathogenic Cedar virus discovered in 2012 ^11^. Moreover, Henipa-like full-length viral sequences were found in African fruit bats ^12^ and Chinese rats (Moijang virus) ^13^. Two major genotypes of NiV have been identified so far: Malaysia and Bangladesh, which share 92% of nucleotide homology ^14, 15^ and present some differences in their pathogenicity ^16^. The NiV genome is composed of a negative-sense, single, non-segmented RNA and contains six transcription units encoding for six viral structural proteins (3′-N-P-M-F-G-L-5′) and three predicted P gene products coding for non-structural proteins, C, V, and W, demonstrated to function as inhibitors of the host innate immune response ^17– 20^.

NiV was first identified as the cause of an outbreak of encephalitis in humans during 1998 to 1999 in Malaysia and Singapore ^21^. The virus has been transmitted from infected pigs to humans, and the control of the epidemic necessitated culling over 1 million pigs, presenting a huge economic burden ^22, 23^. Although no further outbreaks have occurred in Malaysia since then, annual outbreaks of the new NiV strain have started since 2001 in Bangladesh ^5^. The new NiV cases have been identified in the other parts of Southeast Asia: one in Philippines ^24^ and three in India, with the last one in the state of Kerala, reaching a fatality rate of 91% ^4^, solidifying NiV as a persistent and serious threat in South Asia.

Fruit bats from *Pteropus* species (flying foxes) have been recognized as the natural host of NiV ^25^. Deforestation in large regions of Southeast Asia damages bat roosting trees and food supplies, leading to the migration of bat colonies toward urban sites, thus increasing the contact with humans ^26, 27^. NiV transmission from bats to humans was shown to occur through consumption of raw date palm juice or fruits contaminated with bat saliva or urine ^28^. Alternatively, transmission occurs via close contact with infected domestic animals acting as viral amplifying vectors, such as pigs or horses, and via inter-human transmission in one third of NiV Bangladesh strain infections ^5, 29, 30^. In addition, NiV and Henipa-like viruses have been molecularly or serologically detected (or both) in *Pteropus* bats in different countries from Asia and Africa ^12^, and the worldwide distribution of these bat species poses a threat to potential NiV pandemics ^7^.

## Nipah virus pathogenesis and animal models

NiV-caused disease is characterized by the onset of non-specific symptoms, including fever, headache, dizziness, vomiting, and myalgia. Later, patients may develop severe encephalitis and pulmonary disease. Respiratory syndrome is observed more frequently in patients infected with NiV Bangladesh. Recently, the persistence of NiV RNA was described in the semen of a patient surviving NiV infection in India ^31^; this is similar to what has been previously reported for Ebola ^32^ and Zika ^33^ virus. Survivors from NiV infection frequently have long-term neurological sequelae ^34^. Furthermore, another clinical syndrome, late-onset encephalitis, has been observed in some patients following an initial NiV infection that was either mild or asymptomatic. Finally, relapse encephalitis could develop as resurgences of the virus, appearing several months to years after recovering from a symptomatic initial infection ^35^, including a case in which encephalitis occurred 11 years after initial infection ^36^.

Primary human epithelial cells from the respiratory tract were shown to be highly permissive to Henipaviruses and may represent the initial site of infection ^37^. Additionally, the virus shows a high neuro-tropism and the ability to infect muscular cells, suggesting rather ubiquitous expression of its entry receptors in different tissues ^2^. In contrast to some other *Paramyxoviridae*, NiV is not lymphotropic, and among different blood cell types, NiV could infect dendritic cells only ^38^. Nevertheless, viral dissemination within the host is facilitated by NiV attachment to circulating leukocytes through binding to heparan sulfate without infecting the cells ^39^, using leukocytes as a cargo allowing viral transfer to endothelial vascular cells through a mechanism of transinfection ^38^.

NiV uses Ephrin-B2 and -B3 as entry receptors that are highly conserved among numerous species ^40– 43^. Indeed, various mammalian species such as hamsters, ferrets, cats and bigger animals, including horses, pigs, and non-human primates, have been experimentally infected and used to develop potential new therapeutics ^44, 45^. Furthermore, *Pteropus* fruit bats, the natural reservoir of the virus, were experimentally inoculated with NiV in order to study their susceptibility to infection, viral distribution, and pathogenesis ^46, 47^. No clinical signs were observed in flying foxes, raising the interest of the scientific community in the study of fruit bat–NiV interactions and understanding their capacity to control NiV infection ^47– 49^.

Infection of hamsters with both NiV or HeV induces acute fatal encephalitis with a pathology similar to that of humans ^50, 51^ and this small-animal model provides a useful tool in studying both pathogenesis and potential countermeasures. As pigs were the critical amplifying host during the NiV outbreak in Malaysia, they have also been used as a model for NiV infection. Indeed, viral shedding, associated with an invasion of the central nervous system, has been associated with a mortality of 10 to 15% in infected animals ^52^. Interestingly, unlike other species, NiV is able to infect certain populations of swine lymphocytes ^53^. Similarly to what has been observed in hamsters ^54^, the NiV Malaysia strain induces higher virus replication and clinical signs in pigs, compared with the NiV Bangladesh strain ^55^. Remarkably, in the ferret model, the NiV Malaysia and Bangladesh strains showed similar pathogenicity ^56^, although higher amounts of viral RNA were recovered in oral secretions from ferrets infected with NiV Bangladesh ^57^.

Although mice represent a small-animal model convenient to study viral infections providing a well-developed experimental toolbox, NiV induces a subclinical infection in elderly wild-type mice only ^58^. However, it has been demonstrated that NiV infection is highly lethal in interferon receptor type I (IFN-I)-deficient mice ^59, 60^.

**Figure 1.**
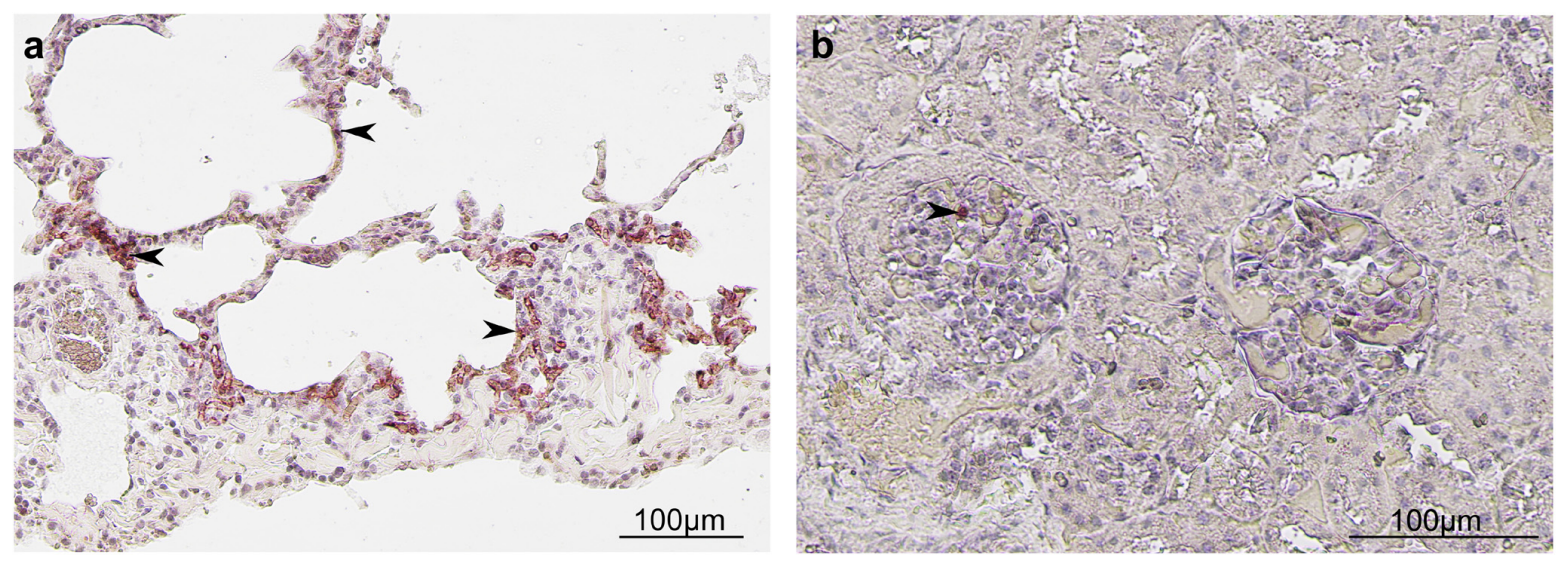
Immunohistochemistry of African green monkey (AGM) tissues after Nipah virus (NiV) infection. An AGM was infected by NiV via the respiratory route, and necropsy was performed 8 days after infection. Immunostaining of lungs ( **a**) and kidney ( **b**) was made by using a polyclonal rabbit antibody specific for NiV nucleoprotein, and hematoxylin was used for the counter-staining. Interstitial pneumonia was found in lungs, inflammatory cells were present in both lungs and kidney, and positive immunostaining for NiV N (arrows) was observed in the alveolar wall and kidney glomerulus.

Development of non-human primate models is particularly important for the advances in anti-viral preventive and therapeutic approaches. Squirrel monkeys ^61^ and African green monkeys (AGMs) ^62^ are susceptible to NiV infection, and the AGM model has been used extensively as its general disease progression and symptomatology are similar to those of NiV-infected humans. NiV infection through the respiratory route in AGM induces a generalized vasculitis and reproduces the clinical symptoms observed in humans, including respiratory distress ^62, 63^, a neurological disease ^64^, and a viral persistence in the brain from surviving animals ^65^. Furthermore, concomitant to human infections, NiV Bangladesh is more pathogenic than the NiV Malaysia strain in AGM ^66^. Pathogenesis following NiV infection is observed mainly in the respiratory tract and is characterized by acute respiratory distress syndrome and pneumonia following infection of epithelial cells ( Figure 1a). As in other animal models, the virus could be found in a wide range of tissues, including kidneys ( Figure 1b), brain, or liver (or a combination of these), suggesting efficient viral dissemination ^62, 67^.

### Innate immunity and interferon type I signaling

**Figure 2.**
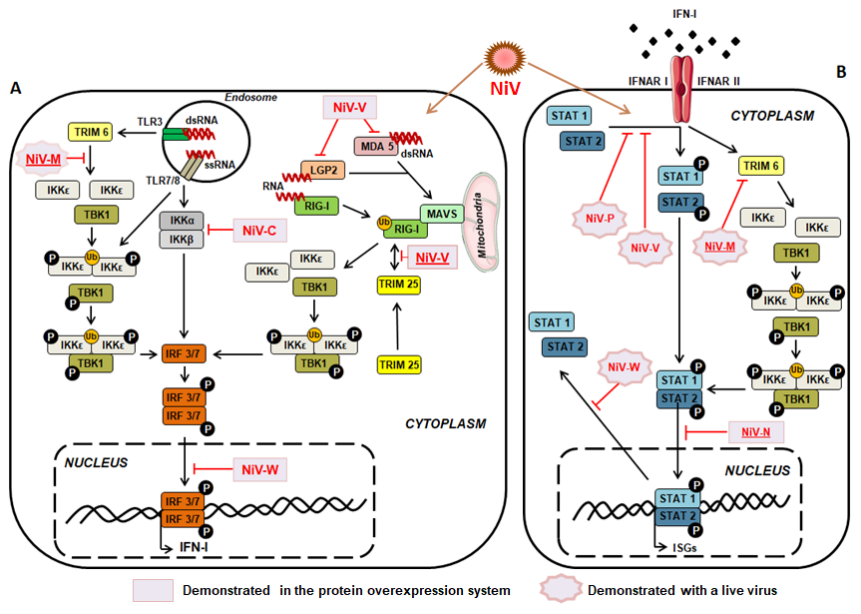
Schematic presentation of Nipah virus (NiV)-induced modulation of type I interferon (IFN-I) production and signaling. ( **a**) NiV infection is followed by the production of viral RNA, which activates TLR and RLR pathways in the cell, leading to the activation of IFN-I and IFN-stimulated genes (ISGs). However, several NiV proteins could interfere with this activation at different levels. NiV-V disrupts MDA5 and LGP2 stimulation and subsequent RIG-I activation ^81^. Conjointly, NiV-C protein counteracts IKKα/β dimerization, important for the activation of IRF3 and IRF7 ^82^, while NiV-W protein prevents nuclear transport of phosphorylated IRF3/7 dimers ^78^. In addition, NiV-V protein inhibits RIG-I activation and its signaling pathway by binding to its caspase activation and recruitment domain (CARD) following anterior binding to TRIM25 that prevents further RIG-I ubiquitination ^80^. Finally, experiments using a live virus NiV deficient in M gene expression highlighted the effect of NiV-M in the degradation of TRIM6 and disruption of IKKε ubiquitination (Ub), oligomerization, and subsequent phosphorylation (P) by preventing synthesis of K48-linked unanchored polyubiquitin chains ^83^. ( **b**) NiV-induced production of IFN-I leads to the stimulation of IFN-I receptor (IFNAR) and subsequent anti-viral signaling, which could be disrupted by several NiV proteins. NiV-N could inhibit nuclear import of STAT1/2 dimer ^84^, while NiV-M triggers degradation of TRIM6 and disrupts subsequent IKKε, TBK1, and STAT1/2 phosphorylation ^83^, as described in (a). In addition, experiments using live virus demonstrated that NiV-P and V could interfere with STAT1 and STAT2 phosphorylation ^73, 75^ while NiV-W prevents their nuclear exportation ^77^. Those mechanisms, combined, constitute the immune evasion strategy displayed during NiV infection, allowing an efficient host invasion. Inhibitory mechanisms presented by underlined NiV proteins correspond to studies published after 2016.

Innate immune response plays a critical role in anti-viral host defense and its modulation during NiV infection has been demonstrated in several reports ^17, 68– 71^. Robust expression of anti-viral genes in lung tissue, including *MX1*, *RSAD2*, *ISG15*, and *OAS1*, during the early stages of NiV infection in ferrets was not sufficient to contain viral dissemination ^56^. Suppression of IFN-I production is known to promote viral spread by disrupting the first lines of defense, resulting in important tissue damage and leading to death. Several mechanisms have been described and both structural and non-structural NiV proteins were found to be involved in the blocking of IFN-I signaling pathway, using distinct strategies ^72– 78^, as summarized in Figure 2.

Inhibition of IFN-I response was observed in different animal models during the course of NiV infection. Indeed, NiV infection of hamsters ^79^ and ferrets ^56^ provides insight into the specific viral signature with a downregulated or delayed IFN-I response during the course of infection. In addition, several *in vitro* studies allowed the identification of viral proteins involved in immune suppression, providing detailed mechanisms of the modulation of IFN-related pathways. Sanchez-Aparicio *et al*. reported interactions between non-structural NiV-V protein and both RIG-I and RIG-I regulatory protein TRIM25 ^80^. They described the binding of the conserved C-terminal domain of NiV-V to caspase activation and recruitment domains (CARDs) of RIG-I and the SPRY domain of TRIM25, thus preventing ubiquitination of RIG-I and its downstream signaling ( Figure 2a). In addition to previously described antagonist effects of NiV-V on MDA5 and STAT1 activation ^73, 81^, this recent report highlights the multirole of NiV-V protein in dismantling the IFN-I response. Furthermore, another study described the capacity of NiV matrix protein (M), known to be important in virus assembly and budding, to disrupt IFN-I signaling ( Figure 2b). Indeed, NiV-M protein interacts with E3-ubiquitin ligase TRIM6, triggering its degradation and subsequent inhibition of IKKε kinase-mediated IFN-I response ^83^. These results were confirmed by a reduced level of endogenous TRIM6 expression upon NiV infection only when M was expressed. Moreover, the role of NiV nucleoprotein (N) was recently reported in hampering IFN-I signaling by preventing the nuclear transport of both signal transducer and activator of transcription 1 (STAT1) and STAT2 ^84^, subsequently impairing the expression of IFN-stimulated genes. All together, these recently described routes used by NiV proteins to prevent host anti-viral response provide new insights into viral evasion mechanisms involved in the control of the IFN-I pathway.

### Adaptive immunity

NiV causes an important modulation of both humoral and cell-mediated immune responses during the course of infection ^85, 86^. The NiV outbreak in Kerala in May 2018 provided the opportunity to study the adaptive immune responses in two surviving patients infected with the NiV Bangladesh strain ^85^. Although absolute number of T-lymphocytes remained normal in blood, the marked elevation of activated CD8 T cells, co-expressing granzyme B and PD-1 was observed, suggesting the increase of lymphocyte population important for the elimination of infected cells. Patients surviving NiV infection also had elevated counts in B-lymphocytes, associated with an important generation of NiV-specific IgM and IgG antibodies. These data support the importance of both humoral and cell-mediated immune responses in the protection against NiV infection. Survivors from NiV infection elicited a stronger, more efficient, and more balanced immune response compared with fatalities.

Three recent studies evaluated immune responses in peripheral blood and tissues in ferrets and monkeys, following infection through the respiratory route ^56, 63, 87^. Analysis of the gene expression profile in ferrets following the infection with the NiV Bangladesh strain showed a time-dependent increase of macrophage markers and an unchanged level of lymphocyte markers in lungs, while brain infection was characterized by limited immune response ^56^, thus presenting the first global characterization of the host gene expression during Henipavirus infection. Study of the peripheral immune response in NiV-infected AGM highlighted the onset of a cell-mediated immune response through the production of Th1-associated cytokines and an increase in CD8 ^+^ T cell activation/proliferation markers in blood, lung, and brain tissues, although neutralizing antibodies were not generated during the 10-day course of infection ^63^. Interestingly, the study of natural killer (NK)-cell response during infection in AGM emphasized an increase in their proliferation, activation, and functional activity during both acute and convalescent phases in surviving animals contrary to succumbing ones ^87^, thus suggesting the implication of NK cells in anti-NiV response.

## New strategies to control Nipah virus infection

Several vaccine development strategies have recently been studied in small-animal models, including chimeric rabies-based ^88^, virus-like particle (VLP)-based ^89^, adenovirus-based ^90^, and epitope-based ^91, 92^ vaccines. Those approaches induced a protection against NiV by triggering a specific response against its envelope glycoprotein G that will require further development using non-human primates to evaluate their efficiency and safety. An additional study using recombinant vesicular stomatitis virus expressing NiV-G protein, in addition to Ebola virus GP protein (rVSV-EBOV-GP-NiV-G), demonstrated complete protection from a high dose of NiV in the hamster model ^93^. That study was followed by further evaluation of the vaccine vector in the AGM model, where the induction of a robust and rapid protective anti-NiV immune response was observed ^94, 95^. Vaccination of animals with rVSV-EBOV-GP-NiV-G vector induced protection against NiV challenge when administered either the day before or at the day of challenge and elicited partial protection when administered up to 1 day post-exposure. A plausible explanation of the mechanism involved in the generation of this fast protection could be the stimulation of the host’s innate immune response, inhibiting viral replication and allowing the development of a virus-specific adaptive immune response. Altogether, this recent work and previous reports highlight the importance of the humoral immune response and the protective role of antibodies directed against viral proteins in the control of NiV infection. Indeed, the human monoclonal antibody specific for Hendra G protein, m102.4, elicited promising results against *Henipavirus* infection following its passive transfer in infected ferrets ^96^ and AGM ^97^ and is being tested in clinical trials.

Although the development of potential NiV vaccines is ongoing and the scrutiny to get authorized vaccines directed against BSL4 pathogens has been accelerated, the only approved vaccine on the market is an animal vaccine directed against HeV in horses in Australia (Equivac-HeV). The importance of a cell-mediated immune protection against Henipaviruses has been demonstrated in hamsters and pigs ^86, 98, 99^, indicating that particular attention should be given to this arm of the immune response for the development of new vaccines. Moreover, these reports underline that both well-balanced innate and adaptive immune responses play important roles in the control of NiV infection.

In parallel to vaccines, other therapeutic strategies have been under development. Recent *in vitro* investigations demonstrated that nucleoside inhibitor 4′-azidocytidine (R1479) and its analogs, previously identified to inhibit flaviviruses, are also capable of inhibiting NiV replication and may present potential broad-spectrum anti-viral candidates for future development ^100, 101^. A recent study in hamsters demonstrated the ability of favipiravir (T-705), a viral RNA-dependent RNA polymerase inhibitor that acts as a purine analog, in preventing NiV-induced morbidity and mortality when administered immediately following infection ^102^. Those results indicate that favipiravir, shown previously to protect against Ebola virus infection ^103^, is a potentially good candidate for post-exposure prophylaxis to NiV. A different approach has been developed by specifically inhibiting NiV entry into the cells by acting on the fusion machinery ^104^. Indeed, viral entry is mediated by the viral envelope glycoproteins G and F (fusion protein) and can be targeted by fusion-inhibitory peptides ^2^. Intra-tracheal administration of these peptides conjugated to lipids, shown to increase their efficiency, was protective in both hamsters and AGM against high-dose lethal NiV challenge. Finally, recent promising work has validated the efficiency of remdesivir (GS-5734), a broad-acting anti-viral nucleotide prodrug, against NiV Bangladesh in an AGM model, demonstrating its ability to protect monkeys if given 24 hours post-infection ^105^. Clinical trials of this drug against Ebola virus have recently been started in democratic republic of Congo ^106^ and a similar approach will be required for the evaluation of remdesivir against NiV.

## Conclusions and future directions

NiV attracts particular attention among members of the *Paramyxovirus* family, as it possesses high zoonotic potential associated with one of the highest fatality rates observed in infectious diseases. The wide distribution of its natural host, the fruit bats, combined with the possibility of the spread of NiV via the respiratory route, raises the risk that pandemics will be caused by this virus in the future and calls for a better understanding of its pathogenesis and the development of efficient anti-viral approaches. NiV proteins were shown to effectively interact with the immune response and disable the establishment of a protective anti-viral immunity. Understanding the host–pathogen relationship at both molecular and cellular levels in different species and elucidating how bats could efficiently control NiV infection represent exciting challenges for future research and may open new avenues in the development of innovative anti-viral strategies. These studies should lead to novel clinical trials, allowing the generation of drugs efficient in the treatment of NiV infection. Further studies require a multidisciplinary approach, putting together virologists, immunologists, epidemiologists, veterinarians, and physicians within a “one health approach” in the common endeavor to understand and control Henipavirus infections.
